# The impact of MFG-E8 in chronic pancreatitis: potential for future immunotherapy?

**DOI:** 10.1186/1471-230X-13-14

**Published:** 2013-01-16

**Authors:** Jan G D’Haese, Ihsan Ekin Demir, Timo Kehl, Jannik Winckler, Nathalia A Giese, Frank Bergmann, Thomas Giese, Markus W Büchler, Helmut Friess, Mark Hartel, Güralp O Ceyhan

**Affiliations:** 1 Department of Surgery, Klinikum Rechts der Isar, Technische Universität München, Ismaninger Str. 22, Munich, D-81675, Germany; 2 Department of General Surgery, University of Heidelberg, Heidelberg, Germany; 3 Institute of Pathology, University of Heidelberg, Heidelberg, Germany; 4 Institute for Immunology, University of Heidelberg, Heidelberg, Germany

**Keywords:** MFG-E8, Chronic pancreatitis, Fractalkine, Fibrosis, Stellate cells, Pain

## Abstract

**Background:**

The glycoprotein MFG-E8 mediates phagocytic clearance of apoptotic cells and influences the pathogenesis and progression of inflammatory diseases. MFG-E8 was shown to attenuate the progression of inflammation and to improve survival in septic rats. Accumulating evidence suggests an immunomodulatory link between MFG-E8 and the pro-inflammatory chemokine fractalkine, which may determine the severity of pain, fibrosis, and inflammation in chronic pancreatitis (CP).

**Methods:**

The expression and localization of MFG-E8 was investigated in CP (n = 62), and normal pancreas (NP; n = 34) by QRT-PCR, Western-blot and immunohistochemistry analyses. Results were correlated with mRNA expression of fractalkine, CX3CR1, and with the presence and degree of pain and fibrosis. Human pancreatic stellate cells (hPSCs) were isolated from CP tissues and evaluated for MFG-E8 mRNA expression after fractalkine stimulation.

**Results:**

MFG-E8-mRNA was significantly overexpressed in CP and isolated hPSCs when compared to NP. Western-blot and immunohistochemistry analysis confirmed accumulation of MFG-E8 in CP, with noticeably increased MFG-E8 immunoreactivity in tubular complexes. MFG-E8 expression correlated significantly with fractalkine expression, severe fibrosis, and the presence of pain in CP patients. Stimulation of hPSCs with fractalkine led to a significant increase in MFG-E8 expression.

**Conclusions:**

In the present study, we demonstrated for the first time that MFG-E8 is significantly up-regulated in CP patients and together with fractalkine correlated noticeably with severe fibrosis and the presence of pain. hPSCs overexpress MFG-E8 upon fractalkine stimulation in vitro, which underlines the suggested immunmodulatory link in CP and may be a key mechanism in CP fibrogenesis and pain generation. Taken together, these novel findings suggest that MFG-E8 blockade may be a promising tool for future immunotherapy in CP to attenuate both fibrosis and pain sensation.

## Background

Chronic pancreatitis (CP) is a chronic inflammatory disease, characterized by a progressive destruction of the pancreatic parenchyma, which often results in severe exocrine and endocrine insufficiency [1,2]. Furthermore, CP is characterized by an impressive infiltration of various subsets of inflammatory cells and severe fibrosis with distinct accumulation of extracellular matrix. Inflammatory cell infiltration in CP is especially striking in intrapancreatic nerves and has been suggested to lead to the neuropathic pain syndrome in CP patients [3]. Moreover, it has been repeatedly shown that inflammatory cells can influence fibrogenesis by supporting the activation of human pancreatic stellate cells (hPSCs), which consequently release ECM proteins leading to fibrosis [4,5]. This activation of hPSCs is driven by the release of cytokines like PDGF, TNFα, and TGFβ from mononuclear cells and leads to a more pro-fibrogenic and pro-inflammatory cell like phenotype of hPSCs. Following activation, hPSCs secrete autocrine factors like periostin and TGFβ which perpetuate their activation and contribute to the vicious cycle of inflammation, fibrosis, and pain in chronic pancreatitis [6].

Milk fat globule epidermal growth factor 8 (MFG-E8) is a glycoprotein which has originally been discovered in milk-fat globules of lactating mice [7]. MFG-E8 contains one epidermal growth factor (EGF)-like domain with an Arg-Gly-Asp (RGD) motif and two tandem c domains (C1 and C2) with homology to discoidin-type lectins and two membrane-binding domains of blood-clotting factors V and VIII [8,9]. MFG-E8 has a signal sequence at the amino-terminus, but no putative hydrophobic membrane-spinning region, suggesting that it is a secreted protein. It binds to cells via its RGD motif, particularly strongly to cells expressing the integrins αvβ3 and αvβ5 [10-12]. MFG-E8 specifically binds to phosphatidylserine exposed on plasma membranes of apoptotic cells and works as a bridging molecule between apoptotic cells and phagocytes, tagging them for directed elimination [13,14].

The localization of MFG-E8 is not limited to inflammatory cells, since it is ubiquitously expressed in different cells and tissue types [15]. It is released by apoptotic endothelial cells which can trigger macrophage reprogramming into an anti-inflammatory phenotype [16]. MFG-E8 has been shown to directly activate proliferation in aortic vascular smooth muscle cells via phosphorylation of ERK1/2 [17]. A recent study by Aziz et al. showed that MFG-E8 attenuates neutrophil infiltration in acute lung injury [18], meaning that MFG-E8 may be able to directly influence the quality of inflammatory cell infiltrations. Moreover, it has been shown that microglia, the phagocytes of the brain, upregulate MFG-E8 upon fractalkine stimulation to label the apoptotic neurons and thereby help them recognize their target cells. Here, again, seems that MFG-E8 works as a bridging molecule between apoptotic cells/neurons and microglia [19]. As in the nervous system, MFG-E8 expression can be induced in peritoneal macrophages of septic rats and mice by the chemokine fractalkine. Elevated fractalkine levels lead to higher MFG-E8 expression and enhanced clearance of apoptotic cells, suggesting a possible novel treatment for patients in sepsis [20]. In this context, we recently demonstrated that pancreatic overexpression of fractalkine in CP is closely linked to visceral pain and to the recruitment of inflammatory cells into the pancreatic tissue and especially to intrapancreatic nerves, with subsequent generation of pancreatic neuritis [21].

By mediating the clearance of apoptotic cells, MFG-E8 attenuates the progression of inflammation and improves survival in septic rats [22,23]. In murine experimental acute colitis, MFG-E8 was down-regulated in the acute phase of colon inflammation, while it gradually became up-regulated during the healing phase [24,25].

Up to now, the potential role and expression of MFG-E8 has never been investigated in CP, especially in regard to possible interactions with the chemokine fractalkine, inflammatory cell infiltration, fibrosis and pain. For this, the expression and localization of MFG-E8 was analyzed in normal pancreas, CP, and in freshly isolated hPSCs from CP tissues and correlated with clinical data and fractalkine expression.

## Methods

### Patients and tissues

A total of 62 CP tissue samples were collected from patients undergoing pancreatic head resection (44 males, 18 females, median age 45 years). The etiology of the pancreatitis was alcoholic CP (n = 30), biliary CP (n = 3), idiopathic CP (n = 29). Diabetes was present in 15 of these 62 patients. All patients were informed verbally, and written informed consent was obtained. Normal pancreatic tissue samples were obtained from healthy organ donors (*n* = 34; 21 male, 13 female; median age 38 years) whenever there was no suitable recipient available. The study protocols were approved by the Ethics Committees of the University of Bern (Switzerland) and the University of Heidelberg (Germany).

The types of surgical resection performed were classical Whipple’s procedure in 7 cases, pylorus-preserving Whipple’s procedure in 8 cases, pancreatic left resection in 4 cases, duodenum-preserving pancreatic head resection (Beger’s procedure) in 24 cases, a duodenum-preserving pancreatic head resection (Bern modification) in 36 cases.

Resected pancreatic tissues were divided into several parts and the aliquots were (a) frozen in liquid nitrogen and stored at −80°C for protein extraction, (b) taken into RNA-later (Ambion, Huntington, UK) for RNA extraction, and (c) fixed in 5% paraformaldehyde and later embedded in paraffin for immunohistochemical analysis.

In all CP patients, the individual pain score was registered before operation, including pain intensity and frequency as described earlier [21,26,27]. The following scale was used to grade the individual intensity of pain: 0 = none, 1 = mild, 2 = moderate and 3 = strong pain. The frequency of pain was graded as 1 = monthly, 2 = weekly and 3 = daily. Pain severity was then calculated by multiplying pain intensity and frequency. According to this pain score, the patients were finally divided into three groups, as described earlier [21,26,27] Pain 0 (0), representing the group of patients who did not have any pain; Pain I (1–3), representing the group of patients with mild pain; and Pain II (4–9), the group of patients who suffered from moderate to severe pain [21,26,27].

### Reagents

Reagents purchased were as follows: DMEM, Trypsin-EDTA, and penicillin-streptomycin from Invitrogen (Karlsruhe, Germany); fetal bovine serum (FBS) from PAN Biotech. For both immunohistochemical and western blot analysis MFG-E8 mouse monoclonal antibody from Santa Cruz Biotechnology (Heidelberg, Germany) was used. For western blot analysis, anti-mouse IgG HRPO-linked antibody and ECL immunoblotting detection reagents were purchased from Amersham Biosciences (Amersham, UK). DAKO Envision system (Hamburg, Germany) was used for immunohistochemistry. All other reagents were from Sigma-Aldrich Chemical Company (Taufkirchen, Germany).

### Immunohistochemistry

Consecutive 3 μm paraffin-embedded tissue sections of both groups were analysed for MFG-E8 immunostaining using the DAKO Envision system (DAKO, Hamburg, Germany), as described previously [26]. Mouse monoclonal MFG-E8 antibody (1:800, Santa Cruz, Heidelberg, Germany) was diluted in DAKO antibody dilutant (DAKO, Hamburg, Germany). Mouse IgG (DAKO, Hamburg, Germany) was used as negative control. Digital imaging was performed with the Zeiss AxioCam HR system (Carl Zeiss AG, Oberkochen, Germany).

### Western blot analysis

Protein extraction and western blot analysis of pancreatic tissues were performed as described previously [26]. A monoclonal mouse MFG-E8 antibody (Santa Cruz, Heidelberg, Germany) was used at the dilution 1:1000 at 4°C overnight. Antibody detection was performed with an enhanced chemiluminescence reaction (Amersham, UK). Equal loading was assured by stripping the blots and probing with the mouse monoclonal anti-GAPDH antibody (Santa Cruz, Heidelberg, Germany). After digital scanning of the western blot films, densitometric analysis was carried out using the open source ImageJ software provided by the National Institutes of Health. Specific signal intensity was calculated and was corrected for the matching equal loading densities as described previously [21].

### Clinicopathological analysis

Consecutive sections obtained from the pancreatic tissue of each patient were stained with hematoxylin and eosin for histomorphological examination. Histopathological analysis was carried out by two independent observers (JGD, IED) blinded to QRT-PCR data, followed by resolution of any differences by joint review and consultation with a third observer (FB). Histomorphological evaluation of the specimens included the severity of fibrosis, which was graded according to a scoring system as described earlier [21]. The severity of fibrosis was revealed by the addition of intralobular and perilobular fibrosis scores as mild 0 (0–4), moderate I (5–9) or severe II (10–12) fibrosis [21]. No pathomorphological abnormalities were detected within the group of normal pancreas.

### Real-time light cycler® quantitative polymerase chain reaction (QRT-PCR)

All reagents and equipment for mRNA/cDNA preparation were purchased from Roche Applied Science (Mannheim, Germany). Extraction of mRNA from normal and CP tissues was prepared by automated isolation using the MagNa Pure LC instrument and isolation kits I (for cells) and II (for tissue). cDNA was prepared using the 1st strand cDNA synthesis kit for RT-PCR according to the manufacturer’s instructions. Quantitative real-time PCR was performed with the Light Cycler Fast Start DNA SYBR Green kit. All primers were obtained from Search-LC (Heidelberg, Germany). The calculated number of specific transcripts were normalized to the housekeeping genes cyclophilin B (CPB) and hypoxanthine guanine phosphoribosyltransferase (HPRT), and expressed as the amount per μl of input cDNA, as described previously [28].

### Isolation and cell culture of human pancreatic stellate cells

Human pancreatic stellate cells (hPSCs) were isolated from freshly resected pancreatic tissue of patients with chronic pancreatitis using the outgrowth method described by Bachem et al. [5]. hPSCs were cultivated in a 1:1 dilution of Ham’s F-12 nutrient medium and low glucose Dulbecco’s modified Eagle’s medium (DMEM) supplemented with penicillin, streptomycin, amphotericin and 10% FBS. To verify the purity of the hPSCs preparation, western blot analysis with primary mouse monoclonal antibody α-SMA (1:100; DAKO Cytomation Hamburg, Germany) was performed.

### Fractalkine stimulation of hPSCs

The isolated hPSCs were grown in six-well plates for RNA extraction. Fresh medium was supplemented with 1% of FBS containing fractalkine (10 and 100 ng/ml) for functional experiments as soon as the cells reached 70% confluence. PBS was used as a control. Changes in MFG-E8 mRNA expression in hPSCs were analyzed in cell lysates after 12, 24 and 48 h using QRT-PCR. All experiments were repeated three times.

### Statistical analysis

Results are expressed as mean ± standard error of the mean (SEM). To compare more than two subgroups of samples with respect to MFG-E8 mRNA levels in tissue samples, the Kruskal-Wallis test was used. When a significant result was found, further pair-wise comparisons of the subgroups were performed by using the Dunn’s post test. The relationship between MFG-E8, fractalkine, CX3CR1, and the degree of fibrosis and pain was examined by using the Spearman-Rho test with the correlation coefficient r and the corresponding p-value. Two-sided p-values were always computed, and an effect was considered statistically significant at a *p*-value ≤ 0.05.

## Results

### MFG-E8 expression in pancreatic tissues

**Figure 1:**
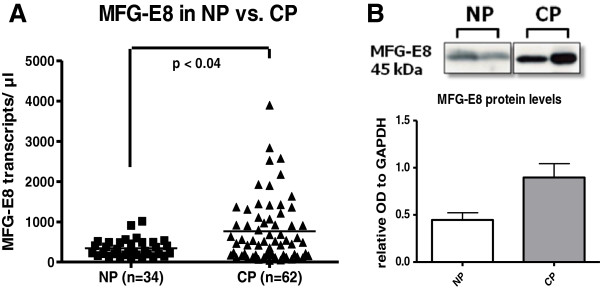
**MFG-E8 in pancreatic tissues.** MFG-E8-mRNA expression, analyzed by QRT-PCR in pancreatic tissues of healthy organ donors (n = 34), compared to chronic pancreatitis patients (n = 62) (**A**). Western blot analysis of MFG-E8 protein levels in pancreatic tissue from CP patients (**B**).

Initially, quantitative RT-PCR analysis was performed to investigate MFG-E8-mRNA expression in normal pancreatic tissue and in chronic pancreatitis. In normal pancreas, the expression of MFG-E8 was 345 ± 37 transcripts/μl, which was significantly increased to 762 ± 97 transcripts/μl (*p* < 0.04) in chronic pancreatitis (Figure 1A). Western blot analysis confirmed the accumulation of MFG-E8 in chronic pancreatitis. MFG-E8 protein levels showed a more than 2-fold increase in chronic pancreatitis compared with normal pancreatic tissues (Figure 1B). MFG-E8 expression did not significantly differ between diabetic or non-diabetic patients (p = 0.32) or in between different etiologies (p = 0.15).

### Immunolocalization of MFG-E8 in normal pancreas and chronic pancreatitis

**Figure 2:**
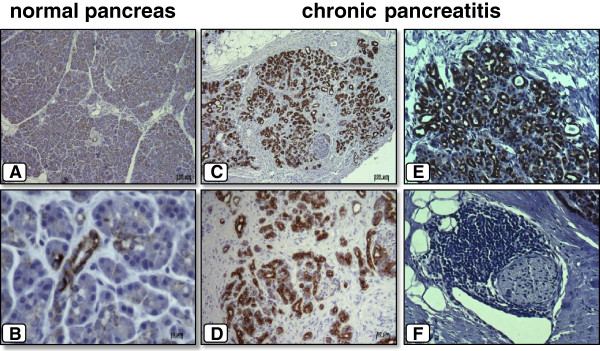
**Immunoreactivity of MFG-E8 in normal pancreas (A + B) and chronic pancreatitis (C - F).** In normal pancreatic tissue samples, immunostaining of MFG-E8 was only moderately present in the ducal and centroacinar cells (**A** + **B**). In chronic pancreatitis, MFG-E8 showed increased immunoreactivity in tubular complexes (**C** - **F**).

To determine the localization of MFG-E8 in the normal pancreas and in chronic pancreatitis, immunohistochemical analysis was performed. In normal pancreatic tissue samples, moderate MFG-E8 immunoreactivity was exclusively evident in ductal and centroacinar cells (Figure 2), whereas vessels, intrapancreatic nerves, and islets did not show any MFG-E8 immunoreactivity (Figure 2). In chronic pancreatitis, MFG-E8 showed increased immunoreactivity in tubular complexes (Figure 2). Interestingly, the extracellular matrix (ECM) itself did not show any immunoreactivity for MFG-E8. However, MFG-E8 immunoreactivity, especially around tubular complexes, was noticeably increased in pancreatic areas neighboring tissue sections with increased fibrosis and ECM (Figure 2C and D).

### Correlation of MFG-E8 with fractalkine and CX3CR1

**Figure 3:**
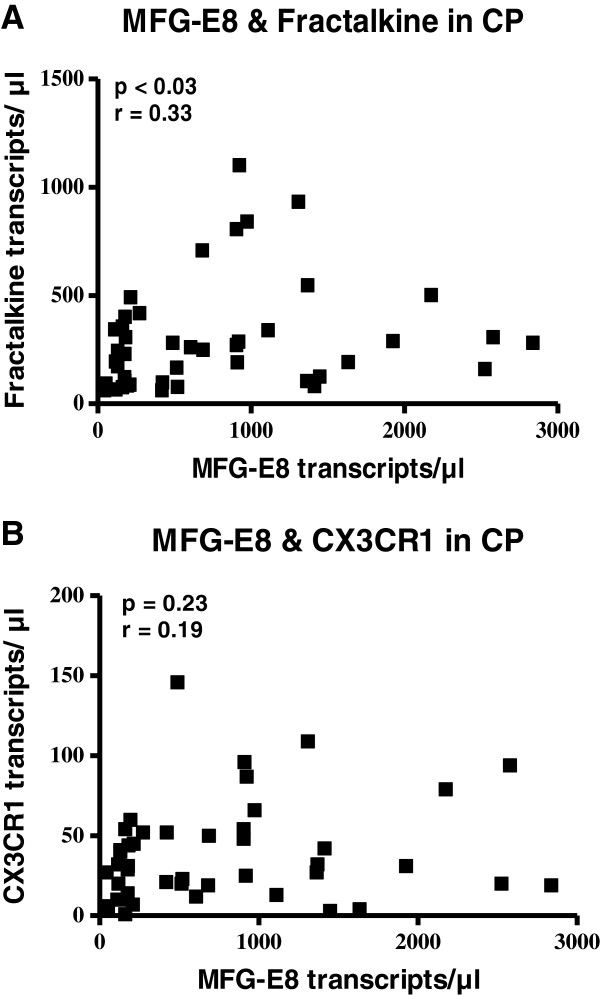
Correlation of MFG-E8 mRNA expression with fractalkine mRNA expression (P < 0.03) (A), and CX3CR1 mRNA expression (B).

To identify possible mediators of MFG-E8, the mRNA expression of fractalkine and CX3CR1 was analyzed by QRT-PCR in all chronic pancreatitis tissue samples and correlated with MFG-E8 mRNA expression. The MFG-E8 mRNA expression correlated positively with fractalkine expression (*p* < 0.03, r-value: 0.33), whereas correlation with its receptor CX3CR1 failed to reach statistical significance (Figure 3A + B).

### MFG-E8 and pancreatic pain

**Figure 4:**
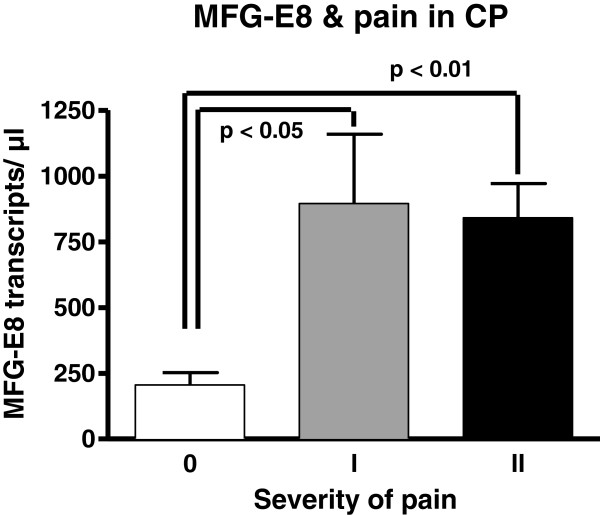
**Relationship of MFG-E8 mRNA expression with the presence/severity of pain in patients with chronic pancreatitis.** Data are expressed as mean ± standard error of the mean (SEM).

Out of the 62 investigated patients, 10 patients had no pain (Pain 0), 11 patients had mild pain (Pain I) and 41 patients had moderate to severe pain (Pain II). Interestingly, patients with no pain showed significantly lower MFG-E8 mRNA levels (206 ± 46 transcripts/μl) than patients with mild (896 ± 263 transcripts/μl; p < 0.05) or moderate to severe pain (841 ± 130 transcripts/μl; p < 0.01; Figure 4).

### The potential impact of MFG-E8 on pancreatic fibrosis in chronic pancreatitis

**Figure 5:**
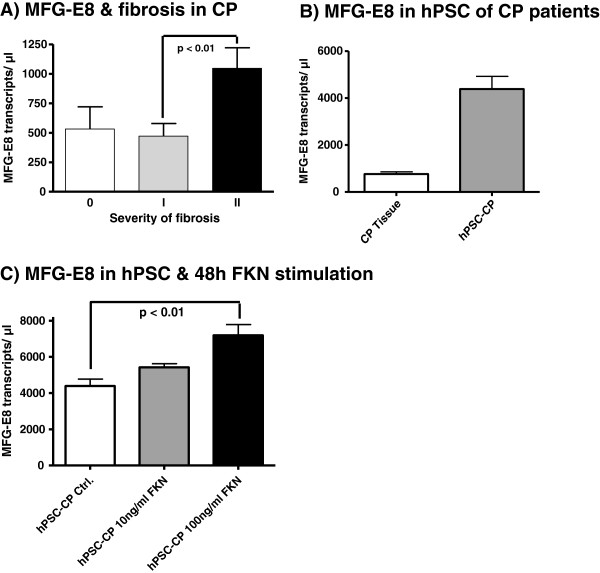
**Relationship of MFG-E mRNA expression with the presence/degree of fibrosis in CP (A).** Comparison of MFG-E8 mRNA expression of human pancreatic stellate cells, isolated from pancreatic tissue of patients with chronic pancreatitis compared to CP tissue as such (**B**). Comparison of MFG-E8 expression in hPSCs 48 hours after stimulation with 10 ng/ml and 100 ng/ml fractalkine compared to unstimulated hPSCs (**C**). Data are expressed as mean ± standard error of the mean (SEM).

To investigate the potential impact of MFG-E8 on pancreatic fibrosis, the severity of fibrosis was determined in every patient and correlated to the MFG-E8 mRNA expression within the CP tissue. MFG-E8 mRNA expression was found to be positively associated with the severity of fibrosis in CP. MFG-E8 expression showed a significant (p < 0.01) increase in the severe fibrosis group II (1046 ± 175 transcripts/μl) compared with the mild fibrosis group I (472 ± 107 transcripts/μl; Figure 5A). To detect the influence of MFG-E8 on fibrogenesis in CP, hPSCs were freshly isolated from human CP resection specimens. Untreated hPSCs from CP tissue showed almost 6 times higher levels of MFG-E8 mRNA (4388 ± 541 transcripts/μl) than the average expression in CP tissue lysates (761 ± 95 transcripts/μl; Figure 5B). In order to assess the putative influence of fractalkine on MFG-E8 expression in these cells, hPSCs where incubated with two different concentrations of recombinant fractalkine. After 12 and 24 hours of fractalkine stimulation QRT-PCR analysis revealed an increasing effect on MFG-E8 expression in hPSCs, but failed to reach a statistical significance (data not shown). However, this increase in MFG-E8 mRNA expression in fractalkine stimulated hPSCs became statistically significant after 48 hours stimulation with 100 ng/ml fractalkine when compared to the control group (7193 ± 1186 transcripts/μl vs. 4388 ± 765 transcripts/μl, p < 0.01; Figure 5C).

## Discussion

MFG-E8 was first identified in the process of phagocytic clearance of apoptotic cells [13]. More recently, it has been shown that MFG-E8 deficient mice show severe inflammatory imbalances, and an MFG-E8-mediated potential therapeutic benefit is evident in experimental inflammatory conditions [18,20,22-25,29-32]. Previous studies have elucidated the role of MFG-E8 in diverse neoplastic and acute inflammatory diseases, yet this is the first study to investigate MFG-E8 expression in chronic pancreatitis.

In the present study, we demonstrated for the first time that MFG-E8 is significantly up-regulated in patients with chronic pancreatitis. Interestingly, and in contrast to acute inflammatory diseases where MFG-E8 is under-expressed in the acute phase and recombinant MFG-E8 seems beneficial, MFG-E8 expression in chronic pancreatitis is significantly higher than in normal pancreatic tissue. Our analysis further revealed that this overexpression is directly related to the presence of pain and associated with severe fibrosis in these patients. The connective link between the chemo-kine fractalkine and MFG-E8 that became evident in chronic pancreatitis further underlines the potential of MFG-E8 as a future therapeutic target in the treatment of painful chronic pancreatitis.

Long-lasting pancreatic pain is the most prominent symptom of chronic pancreatitis which has recently been suggested to be a mixed-type pain from both nociceptive and neuropathic mechanisms [33]. Previous studies have demonstrated a key correlation between the extent of neural damage, neural plasticity, neural inflammatory cell infiltration (pancreatic neuritis), and the pain frequency and intensity of CP patients, thereby underlining the importance of neuropathic mechanisms in CP pain generation [27,34]. In this study we were able to demonstrate that MFG-E8 correlates significantly with the presence of pancreatic pain in CP patients. This observed correlation may theoretically be explained by three different possible pathways: a) MFG-E8 expression may be induced by a yet unknown common factor that likewise induces pain in CP patients, b) MFG-E8 may have an analgesic potential and is therefore released by CP tissue as a reaction on a pain stimulus in the attempt to limit pain sensation. While these possibilities can currently not be completely ruled out, it seems more likely that c) MFG-E8 may influence the extent of neural alterations in chronic pancreatitis and thereby aggravate chronic pain sensation in these patients. While the exact mechanisms remain to be unravelled, a direct negative influence of MFG-E8 on neural inflammatory cell infiltration seems unlikely since MFG-E8 has been shown to rather attenuate neutrophil infiltration in acute lung injury [18]. It is more likely that MFG-E8 modulates pain sensations by directly aggravating the extent of neural damage, possibly by MFG-E8 mediated phagocytosis of viable neurons and/or glial cells as it has very recently been shown for states of neuroinflammation [35]. Accordingly, and consistent with findings in cancer therapy, MFG-E8 blockade - not treatment with recombinant MFG-E8 - may be a possible treatment for chronic pancreatic pain. This idea seems especially tempting since MFG-E8, as a secreted protein, may be rather easily targeted by appropriate blocking antibodies.

Our understanding of the cellular and molecular events leading to the development of pancreatic fibrosis has improved significantly in recent years, largely due to the isolation and characterization of pancreatic stellate cells (PSCs) which play a key role in fibrogenesis [5,36]. Stellate cells are resident cells of the pancreas, located at the basolateral aspect of acinar cells. During inflammatory injury, PSC become activated by migrating mononuclear inflammatory cells to produce and secrete extracellular matrix proteins such as collagen, fibronectin, and laminin, but also pro-inflammatory cytokines and chemokines [37-40]. Our data show that MFG-E8 is highly expressed in human pancreatic stellate cells isolated from chronic pancreatitis tissue, suggesting that MFG-E8 is secreted in high levels by activated hPSC in the chronic state of pancreatitis. On the other hand, high MFG-E8 expression may be in indicator for enhanced hPSC turnover in chronic pancreatitis with a higher rate of phagocytosis of dying hPSCs. This is further underlined by our immunohistological findings that MFG-E8 immunoreactivity was noticeably increased in pancreatic areas neighboring tissue sections with increased fibrosis and ECM. While the ECM itself showed little to no immunoreactivity for MFG-E8 in our study, neighboring regions which are known to contain the activated pancreatic stellate cells showed the highest immunoreactivity [6]. Furthermore, we were able to show that MFG-E8 expression significantly correlated with severe fibrosis in CP patients. According to our observed correlations with pain this correlation as such may be explained by different possible mechanisms. The most likely however is that MFG-E8 may aggravate fibrogenesis in CP patients, possibly by direct modulation of hPSC activation. This MFG-E8 overexpression in hPSC may be on the one hand an indicator of apoptosis, but on the other hand indicate enhanced growth signaling, as MFG-E8 was previously shown to induce PDGF signaling and regulate cyclin D1/D3 expression, especially in neoplastic cells. However, since smoking habits were unfortunately not recorded systematically in the medical history reports we cannot entirely exclude smoking as a systemic confounder influencing MFG-E8 expression in CP.

Another important finding of our study is the strong correlation of the expression of MFG-E8 and the chemokine fractalkine. Fractalkine is the sole member of the fourth class of chemokines and is known to mediate both chemotaxis and adhesion [41]. Fractalkine can be released by proteolysis at a membrane-proximal region by TNF-α converting enzyme (TACE, ADAM17) and ADAM10 [42,43]. It was furthermore reported to be involved in the pathogenesis and progression of numerous inflammatory diseases including chronic pancreatitis [21,44-48]. Recent investigations by our group revealed that the expression of fractalkine strongly correlates with the degree of fibrosis, the severity of neural inflammatory cell infiltration (“pancreatic neuritis”), and the severity and frequency of pain in chronic pancreatitis [21]. The correlation of MFG-E8 expression with fractalkine may therefore suggest an equally important role of MFG-E8 in chronic pancreatitis. This assumption is underlined by the fact that MFG-E8 was likewise significantly correlated to the presence of pain and severe fibrosis in chronic pancreatitis. At last, fractalkine expression seems to be closely linked to MFG-E8 expression since fractalkine stimulation of peritoneal macrophages and microglia has been reported to lead to MFG-E8 overexpression [19,20]. Likewise, we were able to show for the first time that isolated human pancreatic stellate cells express MFG-E8 and show a significant increase in MFG-E8 expression after fractalkine stimulation. We therefore could illustrate that the connective link between MFG-E8 and fractalkine, which has been shown for microglia and macrophages in acute inflammatory conditions is similarly evident in hPSC in chronic pancreatitis. This connective link again underlines the significance of MFG-E8 as a potential therapeutic target.

## Conclusion

In conclusion, our novel findings demonstrate that MFG-E8 is overexpressed in chronic pancreatitis and that MFG-E8 expression is closely linked to expression of the chemokine fractalkine. Isolated hPSCs, which are thought to play a key role in fibrogenesis, showed especially high expression of MFG-E8 and even further increased their MFG-E8 expression upon fractalkine stimulation. MFG-E8 expression is moreover strongly associated with the presence of pain and severe fibrosis, making MFG-E8 a promising candidate for future investigations in regards to its potential as a novel therapeutic target in future treatment of painful chronic pancreatitis.

## Competing interests

The authors declare that they have no competing interests

## Authors’ contributions

JGD carried out the immunohistochemical studies and the histopathological analysis, participated in the design of the study and drafted the manuscript. IED carried out the statistical analysis, the histopathological analysis and helped to draft the manuscript. TK and JW carried out the western blot analysis and the cell culture experiments. NAG and TG performed the QRT PCR analysis and interpretation. FB helped to carry out the histopathological analysis as a third observer. MWB and HF participated in the design and coordination of the study. MH and GOC conceived and coordinated the study, participated in its design, and majorly helped to draft the manuscript. All authors read and approved the final manuscript.

## Pre-publication history

The pre-publication history for this paper can be accessed here:

http://www.biomedcentral.com/1471-230X/13/14/prepub
